# Spectrum of Cervical Insufficiency: Management Strategies from Asymptomatic Shortening to Emergent Membrane Prolapse

**DOI:** 10.3390/jcm14238506

**Published:** 2025-11-30

**Authors:** Dimitris Baroutis, Eleni Katsianou, Ioannis Fragiskos, Maria-Eleni Papakonstantinou, Konstantinos Koukoumpanis, Aikaterini-Gavriela Giannakaki, Alexander A. Tzanis, Vasilios Pergialiotis, Michael Sindos, George Daskalakis

**Affiliations:** 1 1st Department of Obstetrics and Gynecology, Alexandra Hospital, National and Kapodistrian University of Athens, 11528 Athens, Greece; 2 MSc in Pediatric Infectious Diseases, National and Kapodistrian University of Athens, 11527 Athens, Greece

**Keywords:** cervical insufficiency, preterm birth, cervical length, cerclage, progesterone, pregnancy loss, miscarriage

## Abstract

**Background/Objectives:** Cervical insufficiency affects 1–2% of pregnancies and represents a significant cause of second-trimester loss and spontaneous preterm birth. This review synthesizes current evidence across the clinical spectrum of cervical insufficiency, providing evidence-based management guidance and identifying areas requiring further investigation. **Methods:** We conducted a comprehensive review of the current literature, evidence-based clinical guidelines, and landmark randomized controlled trials examining diagnostic frameworks, therapeutic interventions, and clinical outcomes across different presentations of cervical insufficiency. Our analysis incorporated data from major obstetric databases, professional society recommendations, and recent comparative effectiveness research. **Results:** Cervical insufficiency diagnosis encompasses three primary categories: history-based, ultrasound-based, and physical examination-based. Vaginal progesterone achieves a 31% reduction in preterm birth before 33 weeks (RR 0.69, 95% CI 0.55–0.88; NNT= 14). Ultrasound-indicated cerclage achieves a 30% relative risk reduction for delivery <35 weeks. The landmark SuPPoRT trial (*n* = 386) demonstrated no statistically significant differences among cerclage, pessary, and progesterone (*p* = 0.4), though formal equivalence trials have not been conducted. Multiple gestations show no benefit from singleton-derived interventions (RR 0.99–1.04). **Conclusions:** Optimal cervical insufficiency management emphasizes individualized approaches based on comprehensive risk stratification and objective cervical assessment, with vaginal progesterone and cervical cerclage serving as cornerstone therapies supported by robust clinical evidence.

## 1. Introduction

Cervical insufficiency, historically termed “incompetent cervix” in earlier literature, represents a complex clinical syndrome characterized by painless cervical dilation leading to recurrent pregnancy loss or spontaneous preterm birth in the absence of regular uterine contractions, clinically evident infection, or other identifiable causes [1,2]. Affecting approximately 1–2% of pregnancies globally, this condition accounts for an estimated 15–20% of recurrent second-trimester pregnancy losses, establishing it as one of the most significant preventable causes of adverse pregnancy outcomes [3,4].

The evolution of cervical insufficiency understanding parallels broader advances in obstetric care and maternal-fetal medicine. Early descriptions from the 1950s employed a binary classification framework, categorizing cervices as either “competent” or “incompetent” based solely on pregnancy maintenance to term [5]. This reductionist approach yielded empirical treatments with inconsistent success and failed to elucidate underlying pathophysiological mechanisms. The paradigm shift from binary classification to recognition of cervical insufficiency as a spectrum disorder evolved throughout the latter twentieth century, propelled by advancing knowledge of cervical physiology, biochemistry, and the intricate mechanisms governing pregnancy maintenance [6,7].

High-resolution transvaginal ultrasonography, introduced in the 1990s, transformed cervical insufficiency diagnosis and management by enabling objective cervical length measurements throughout gestation [8,9]. This technological breakthrough facilitated the transition from subjective clinical assessment to evidence-based decision-making anchored in quantitative cervical evaluation. The seminal epidemiological investigation by Iams and colleagues established the critical relationship between cervical length and spontaneous preterm birth risk, demonstrating that cervical shortening typically antedates clinical manifestations by weeks to months, thereby creating actionable windows for preventive intervention [10].

The burden of cervical insufficiency extends beyond immediate medical costs to include lifelong consequences of preterm birth. These include cerebral palsy, neurodevelopmental delays, chronic respiratory disease, visual and hearing impairments, and complications requiring extensive medical care and family resources [11,12]. Economic analyses from developed healthcare systems demonstrate that each case of severe preterm birth prevented through effective cervical insufficiency management yields savings of hundreds of thousands of dollars in immediate neonatal intensive care costs and protracted developmental support services [13].

### Epidemiology and Population Demographics

The epidemiology of cervical insufficiency varies significantly across different populations, geographic regions, and healthcare systems, reflecting complex interactions among genetic factors, environmental influences, healthcare access patterns, and methodological differences in diagnosis and reporting [14,15]. Population-based studies from developed countries consistently report prevalence rates ranging from 0.5% to 2.0% of all pregnancies, with higher rates observed in specific high-risk populations including women with prior preterm births, history of cervical procedures, or underlying connective tissue disorders [16,17].

Racial and ethnic disparities in cervical insufficiency outcomes have been consistently documented across multiple healthcare systems, with African American women experiencing disproportionately higher rates of preterm birth and associated complications compared to other demographic groups [18,19]. These disparities likely reflect multifactorial causation involving genetic predisposition, environmental exposures, social determinants of health, including access to quality prenatal care, and systemic healthcare inequities that influence both prevention and treatment effectiveness [20,21].

Advanced maternal age represents an increasingly important epidemiological consideration as reproductive patterns shift toward delayed childbearing in developed societies [22]. Women over 35 years demonstrate increased risks of cervical insufficiency, particularly when combined with other risk factors such as prior cervical procedures or multiple gestations achieved through assisted reproductive technologies [23,24].

## 2. Methods

### 2.1. Literature Search Strategy

A comprehensive literature search was conducted using PubMed/MEDLINE, Embase, Scopus, and the Cochrane Library from database inception through October 2024. The search strategy employed the following key terms in various combinations: “cervical insufficiency,” “cervical incompetence,” “short cervix,” “cerclage,” “cervical pessary,” “progesterone,” “preterm birth prevention,” and “cervical length.” Boolean operators (AND, OR, NOT) were used to refine searches.

### 2.2. Inclusion and Exclusion Criteria

Studies were included if they: (1) were published in English; (2) comprised randomized controlled trials (RCTs), meta-analyses, systematic reviews, large prospective cohorts, or authoritative clinical guidelines; (3) addressed singleton or multiple gestations with cervical insufficiency or short cervix; and (4) evaluated management strategies including cerclage, pessary, progesterone, or expectant management. Studies were excluded if they: (1) were case reports or small case series (*n* < 20); (2) lacked adequate methodological description; or (3) focused exclusively on non-cervical causes of preterm birth.

### 2.3. Quality Assessment and Evidence Synthesis

Evidence quality was assessed using the Grading of Recommendations Assessment, Development and Evaluation (GRADE) framework for interventional studies. Clinical practice guidelines from the American College of Obstetricians and Gynecologists (ACOG), Society for Maternal-Fetal Medicine (SMFM), National Institute for Health and Care Excellence (NICE), and International Society of Ultrasound in Obstetrics and Gynecology (ISUOG) were incorporated.

### 2.4. Classification

This manuscript represents a narrative review with systematic search methodology, designed to provide a clinically oriented synthesis of current evidence and practical management algorithms for cervical insufficiency across its clinical spectrum.

## 3. Pathophysiology and Risk Factors

### 3.1. Molecular Mechanisms and Cervical Remodeling

The cervix undergoes remarkable structural and biochemical transformations throughout pregnancy, involving precisely coordinated remodeling of extracellular matrix components to maintain structural integrity while preparing for eventual parturition [25,26]. Understanding these complex physiological processes is essential for understanding the pathological mechanisms underlying cervical insufficiency and developing targeted therapeutic interventions.

The human cervix consists primarily of extracellular matrix components, with collagen comprising approximately 80% of dry tissue weight, predominantly types I and III collagen providing tensile strength and structural support [27]. Proteoglycans, particularly dermatan sulfate and hyaluronic acid, contribute to tissue hydration and biomechanical properties, while elastin fibers provide elasticity and resilience [28]. Smooth muscle cells, constituting approximately 10–15% of cervical tissue, contribute to active contractile function and respond to hormonal influences throughout pregnancy [29].

During normal pregnancy, the cervix maintains a delicate balance between structural integrity necessary for fetal support and progressive softening required for eventual delivery [30]. This remodeling process involves complex molecular cascades including matrix metalloproteinase activation, collagen degradation and synthesis, inflammatory mediator release, and hormonal modulation of tissue properties [31,32]. The precise timing and extent of these changes determine cervical competence and pregnancy outcome.

Cervical insufficiency results from disruption of normal remodeling processes, leading to premature cervical ripening and inability to withstand the increasing mechanical stresses associated with advancing pregnancy, growing fetal weight, and amniotic fluid accumulation [33,34]. Multiple pathophysiological pathways can contribute to this dysfunction, including chronic inflammatory processes, matrix metalloproteinase imbalances, genetic factors affecting collagen synthesis and degradation, hormonal deficiencies particularly involving progesterone signaling, and mechanical factors related to prior cervical trauma or congenital structural abnormalities [35,36].

Recent research has identified specific inflammatory mediators and molecular pathways involved in pathological cervical remodeling, including interleukin-1β, tumor necrosis factor-α, and prostaglandin E2 signaling cascades [37,38]. These inflammatory processes can be triggered by subclinical infections, mechanical stress, hormonal changes, or genetic predisposition, leading to accelerated collagen degradation and premature cervical softening.

### 3.2. Risk Factors and Clinical Associations

Understanding underlying risk factors for cervical insufficiency is essential for identifying high-risk patients, implementing appropriate surveillance strategies, and optimizing preventive interventions [39,40]. Risk factors can be systematically categorized into congenital and acquired conditions, each with distinct pathophysiological mechanisms and implications for clinical management.

#### 3.2.1. Congenital Risk Factors

Congenital risk factors include structural uterine anomalies such as unicornuate or bicornuate uteri, which may be associated with cervical developmental abnormalities affecting structural integrity [41]. In utero diethylstilbestrol exposure, now primarily of historical significance, caused characteristic cervical and uterine structural abnormalities that significantly increased cervical insufficiency risk [42]. Genetic disorders affecting connective tissue integrity, particularly Ehlers-Danlos syndrome, Marfan syndrome, and other collagen disorders, predispose to cervical insufficiency through fundamental defects in extracellular matrix composition and biomechanical properties [43,44].

#### 3.2.2. Acquired Risk Factors

Acquired risk factors encompass a broader range of clinical scenarios with varying degrees of cervical insufficiency risk. Prior cervical trauma from mechanical dilation during pregnancy termination procedures, particularly when performed using larger dilators or in the setting of advanced gestational age, can cause direct structural damage to the internal cervical os and surrounding tissues [45,46]. The risk appears related to the degree of mechanical dilation required and the skill and technique of the proceduralist.

Cervical excisional procedures for cervical dysplasia treatment represent the most extensively studied and clinically significant acquired risk factor for subsequent cervical insufficiency [47,48]. Cold knife conization, particularly when involving large tissue volumes or deep excision extending beyond 1.5–2.0 cm, demonstrates the strongest association with subsequent pregnancy complications [49,50]. Loop electrosurgical excision procedures and laser conization carry intermediate risks proportional to the volume and depth of tissue removed [51,52]. The mechanism involves direct removal of cervical structural support combined with healing-related fibrosis that may compromise biomechanical properties [45,46,47,48,49,50,51,52].

## 4. Contemporary Diagnostic Framework

### 4.1. Evidence-Based Classification System

Contemporary approaches to cervical insufficiency diagnosis have evolved into sophisticated, evidence-based frameworks that acknowledge the condition’s spectrum nature while providing clear, actionable criteria for clinical decision-making [53,54]. The American College of Obstetricians and Gynecologists, in collaboration with the Society for Maternal-Fetal Medicine, has established three primary diagnostic categories, each validated through extensive clinical research and supported by robust outcome data from multiple international trials [55,56]. Table 1 presents the comprehensive diagnostic framework with corresponding evidence levels and management recommendations.

This classification system represents a major advance in clinical practice by providing standardized criteria that facilitate consistent diagnosis, appropriate patient selection for interventions, and meaningful comparison of outcomes across different healthcare settings and research studies [57,58].

### 4.2. Ultrasonographic Assessment Standards

Transvaginal ultrasonography represents the gold standard for objective cervical assessment, providing reproducible measurements that guide critical clinical decisions regarding intervention timing and intensity [59,60]. Standardized measurement techniques and interpretation criteria are essential for consistent, reliable results across different practitioners, institutions, and healthcare systems [61].

#### Technical Standardization

Optimal cervical length assessment requires adherence to specific technical protocols to ensure measurement accuracy and reproducibility [50,51,52,53,54,55,56,57,58,59,60,61,62,63]. The patient should have an empty bladder to avoid artificial cervical compression, and should be positioned in dorsal lithotomy position with comfortable hip flexion to facilitate probe placement [64]. The transvaginal probe should be inserted gently to avoid cervical compression that can artificially elongate the apparent cervical length, and excessive pressure should be avoided throughout the examination [65].

The cervical canal should appear as a distinct hypoechoic line extending from the internal cervical os to the external cervical os, with clear visualization of both anatomical landmarks required for accurate measurement [66,67]. Measurement involves placement of electronic calipers at the internal and external os endpoints, following the curvature of the cervical canal when present. The shortest measurement obtained from three separate acquisitions is typically used for clinical decision-making [68].

Dynamic assessment during the ultrasound examination provides additional valuable information about cervical competence and functional integrity [69]. Gentle fundal pressure or asking the patient to cough can reveal cervical funneling or dynamic shortening not apparent during resting measurements [70]. The presence, extent, and persistence of cervical funneling provide important prognostic information and may influence intervention decisions even when overall cervical length remains within normal ranges [71].

## 5. Management of Asymptomatic Short Cervix

### 5.1. Vaginal Progesterone Therapy

Vaginal progesterone is a cornerstone intervention for asymptomatic cervical shortening, supported by evidence from multiple randomized controlled trials, systematic reviews, and meta-analyses [72,73]. The therapeutic rationale builds on extensive research demonstrating progesterone’s essential role in maintaining pregnancy through multiple complementary mechanisms including uterine smooth muscle relaxation, anti-inflammatory effects, and preservation of cervical structural integrity [74,75].

The landmark study by Fonseca et al. represented a pivotal advancement in cervical insufficiency management, demonstrating a statistically significant 44% reduction in spontaneous preterm birth before 34 weeks of gestation in patients with cervical length ≤ 15 mm treated with 200 mg micronized progesterone suppositories compared to placebo [76]. This trial provided the first robust evidence that pharmacological intervention could effectively prevent preterm birth in women with documented cervical shortening.

The PREGNANT (Progesterone for the Prevention of Preterm Birth in Women with a Short Cervix) trial further validated progesterone efficacy using a different formulation and broader inclusion criteria [77]. This multicenter, randomized, double-blind, placebo-controlled trial enrolled 458 women with cervical length 10–20 mm and demonstrated significant reductions in preterm birth rates across multiple gestational age thresholds using 90 mg progesterone gel daily.

A comprehensive individual patient data meta-analysis incorporating data from multiple international trials provided the highest level of evidence available, demonstrating statistically significant reductions in both preterm birth rates and composite neonatal morbidity and mortality outcomes [78]. This analysis included over 970 women from five randomized trials and confirmed progesterone’s efficacy across different populations, formulations, and clinical settings.

#### Mechanisms of Progesterone Action

Progesterone exerts multiple beneficial effects through diverse molecular and physiological mechanisms that collectively maintain pregnancy and prevent premature cervical ripening [79,80]. At the cellular level, progesterone maintains uterine smooth muscle quiescence through modulation of gap junction proteins, calcium channel activity, and contractile protein expression [81]. Anti-inflammatory mechanisms represent another crucial aspect of progesterone’s therapeutic action, particularly relevant given the role of inflammatory processes in pathological cervical remodeling [82,83]. Direct effects on cervical extracellular matrix composition and remodeling represent a third important mechanism through which progesterone prevents cervical insufficiency [84].

## 6. High-Risk Population Management

### 6.1. Ultrasound-Indicated Cerclage

For patients with prior spontaneous preterm birth who develop cervical shortening ≤ 25 mm before 24 weeks of gestation, ultrasound-indicated cerclage represents the preferred therapeutic approach based on robust evidence from multiple randomized trials and meta-analyses [85,86]. This strategy exemplifies precision medicine principles by linking therapeutic intervention directly to objective evidence of cervical dysfunction rather than applying interventions universally to all high-risk patients.

Meta-analytic evidence demonstrates that ultrasound-indicated cerclage placement reduces preterm birth risk compared to expectant management, with relative risk reductions of approximately 30% for preterm birth < 35 weeks of gestation [87]. These benefits appear most pronounced for preterm birth < 32 weeks, where the absolute risk reductions are largest and the clinical significance of prevention is greatest [88].

The precision medicine approach of cervical length screening with ultrasound-indicated cerclage achieves pregnancy outcomes comparable to prophylactic history-indicated cerclage while avoiding unnecessary procedures and their associated risks in approximately 60% of high-risk patients who maintain normal cervical length throughout pregnancy [89,90].

### 6.2. History-Indicated Cerclage

History-indicated cerclage remains an appropriate and effective intervention for patients with classic histories of recurrent second-trimester pregnancy losses preceded by painless cervical dilation [91]. This approach involves prophylactic cerclage placement between 12–16 weeks of gestation based solely on obstetric history without requiring evidence of current pregnancy cervical abnormalities [92].

Success rates for history-indicated cerclage range from 70–90% depending on patient characteristics, severity of prior losses, surgical technique, and definition of successful outcomes [93]. The McDonald technique involves placement of a purse-string suture around the cervix at the level of the internal os and is generally easier to perform and remove [94]. The Shirodkar technique involves dissection of the bladder and placement of suture at the internal os level, potentially providing stronger mechanical support but requiring more extensive surgical dissection [95].

### 6.3. Combination Therapy

Recent evidence, predominantly derived from meta-analyses including observational cohort studies rather than randomized controlled trials specifically designed to evaluate combined therapy, suggests that combining vaginal progesterone supplementation with cervical cerclage may provide additive benefits for high-risk patients [96]. A systematic review and meta-analysis by Aubin et al. including eight studies with 668 participants demonstrated that combined therapy results in significantly lower preterm birth rates at <37 weeks compared to cerclage alone (RR 0.51, 95% CI: 0.37–0.79) or progesterone alone (RR 0.75, 95% CI: 0.58–0.96), with a number needed to treat of 4 (95% CI: 3–25) to prevent one preterm birth < 37 weeks [96]. The benefit extended across multiple gestational age thresholds, with combined therapy showing significant reductions in preterm birth at <34 weeks (RR 0.47, 95% CI: 0.31–0.72), <32 weeks (RR 0.45, 95% CI: 0.29–0.69), and <28 weeks (RR 0.34, 95% CI: 0.12–0.98) when compared to cerclage alone [96]. Additionally, combined therapy was associated with decreased neonatal mortality, increased birthweight, increased gestational age at delivery, and a longer latency period between intervention and delivery compared to single-agent therapy. The most consistent benefit has been observed in singleton pregnancies with cervical length < 25 mm or prior spontaneous preterm birth, with the therapeutic effect appearing most pronounced in ultrasound-indicated cerclage populations.

Large retrospective cohort data provide further support for these findings, demonstrating reduced rates of spontaneous preterm birth with combined therapy, particularly in ultrasound-indicated and examination-indicated cerclage populations [97]. The multicenter international retrospective cohort study by Tolosa et al., encompassing diverse clinical settings, confirmed that concurrent progestogen and cerclage therapy was associated with significant pregnancy prolongation and improved perinatal outcomes in high-risk women [97]. Subgroup analyses within this cohort suggested that the strength of evidence for benefit appeared greatest for women receiving ultrasound-indicated or physical examination-indicated cerclage in combination with vaginal progesterone, as opposed to history-indicated cerclage alone. However, the quality of evidence remains moderate, as most included studies were observational in nature with inherent risk of selection bias and confounding by indication. The absence of adequately powered randomized controlled trials specifically designed to evaluate the synergistic effects of combined therapy limits definitive conclusions regarding optimal patient selection and timing of interventions. Further prospectively designed randomized controlled trials with standardized protocols are urgently needed to confirm these promising findings, establish evidence-based guidelines for clinical implementation, and identify specific patient populations most likely to benefit from dual therapy. Table 2 presents comprehensive comparative effectiveness data for various interventions in high-risk patients.

## 7. Comparative Effectiveness Evidence

### 7.1. The SuPPoRT Trial

The SuPPoRT (Stitch, Pessary, or Progesterone Randomised Trial) represents the first direct randomized comparison of cervical cerclage, pessary, and vaginal progesterone for preventing preterm birth in women with short cervix [98]. This multicenter equivalence trial enrolled 386 women with singleton pregnancies and transvaginal ultrasound-documented cervical length < 25 mm between 14^+0^ and 23^+6^ weeks’ gestation across 19 UK obstetric units. The study population was predominantly at high risk: 85% had prior risk factors for preterm birth, 39% had experienced prior spontaneous preterm birth or mid-trimester loss, and 46% had undergone prior cervical surgery [98].

The trial demonstrated equivalent efficacy across all three interventions for the primary outcome of preterm birth before 37 weeks of gestation (cerclage 30.5%, pessary 31.2%, progesterone 24.2%; overall *p* = 0.4), with all pairwise risk differences crossing zero and falling within the prespecified 20% equivalence margin: cerclage versus pessary risk difference was −0.7% (95% CI −12.1 to 10.7), cerclage versus progesterone was 6.2% (95% CI −5.0 to 17.0), and progesterone versus pessary was −6.9% (95% CI −17.9 to 4.1) [98]. No significant differences were observed in secondary outcomes including preterm birth before 34 weeks (18.1%, 18.0%, 16.7%, respectively; *p* = 0.9) or composite adverse perinatal outcome (8.6%, 7.2%, 12.1%; *p* = 0.4). Importantly, emergency cerclages for bulging membranes were required in 0% of cerclage group versus 4.1% of the pessary group and 7.7% of the progesterone group (*p* < 0.001), indicating that post-intervention cervical surveillance remains critical regardless of initial treatment choice [98].

These findings establish Level A evidence that cerclage, pessary, and vaginal progesterone demonstrate comparable effectiveness in women with mid-trimester short cervix. This shifts clinical practice toward individualized shared decision-making based on patient preferences, local expertise, side effect profiles, and contraindications rather than hierarchical intervention selection [98,99,100]. The trial’s pragmatic design and high external validity—including protocol deviations reflective of real-world clinical practice where 15–19% of women received alternative treatments—enhances the generalizability of results to diverse clinical settings.

### 7.2. Cervical Pessary Evidence

The clinical development of cervical pessary devices exemplifies both promise and challenges of therapeutic innovation [101]. Initial enthusiasm from the PECEP trial, reporting 82% reduction in preterm birth before 34 weeks [102], was not confirmed by subsequent larger trials. The TOPS trial (n = 544) demonstrated no reduction in preterm birth before 37 weeks (pessary 45.5% vs. usual care 45.6%; RR 1.00, 95% CI 0.83–1.20), but documented increased fetal or neonatal/infant death (13.3% vs. 6.8%; RR 1.94, 95% CI 1.13–3.32; NNH 15) [103]. The ProTWIN trial (n = 813 twin pregnancies) showed no overall benefit (RR 0.98, 95% CI 0.69–1.39), though a subgroup with cervical length <38 mm demonstrated reduced preterm birth before 32 weeks (11% vs. 25%; RR 0.44, 95% CI 0.20–0.98) [104]. Based on accumulating evidence, current guidelines recommend against routine pessary use for preventing preterm birth [105,106].

## 8. Emergency and Advanced Presentations

### Emergency Cerclage

Emergency presentations of cervical insufficiency represent the most challenging scenarios in contemporary obstetric practice, characterized by advanced cervical dilation often with visible fetal membranes protruding through the cervical os [90,107]. Emergency or rescue cerclage represents a last-resort intervention for carefully selected patients who might otherwise deliver at previable or extremely preterm gestational ages [108].

Optimal candidates include patients presenting between 14–24 weeks of gestation with cervical dilation of 2–6 cm, minimal uterine contractile activity, intact or limited membrane prolapse, and absence of clinical evidence of intraamniotic infection [109,110]. Contraindications include established intraamniotic infection, active vaginal bleeding, advanced labor, fetal death or lethal anomalies, and severe maternal medical conditions [111].

The systematic exclusion of intraamniotic infection represents the most critical component of emergency cerclage evaluation [112]. Amniocentesis is strongly advised when feasible and clinically safe, in situations of cervical dilation over 2–3 cm to obtain amniotic fluid for microbiological and biochemical analysis [113,114]. Meta-analytic evidence suggests that emergency cerclage can achieve overall fetal survival rates of approximately 73% compared to 36% with expectant management alone [115]. Table 3 presents detailed outcomes stratified by clinical presentation characteristics.

## 9. Multiple Gestations

### Distinct Pathophysiology

The management of cervical shortening in multiple gestations presents fundamentally different pathophysiological and clinical challenges compared to singleton pregnancies [117,118]. The pathophysiology of preterm birth in multiple pregnancies relates primarily to uterine overdistention and increased mechanical stress rather than primary cervical structural weakness [119].

Extensive research has evaluated interventions in multiple gestations with consistently disappointing results [120,121]. Vaginal progesterone (PREDICT trial, n = 500) showed no benefit: composite adverse neonatal outcome 20.0% vs. 19.4% (RR 1.03, 95% CI 0.67–1.58) [122]. Similarly, 17-OHPC in 661 twin pregnancies demonstrated no efficacy: preterm birth <35 weeks 41.5% vs. 39.7% (RR 1.02, 95% CI 0.81–1.28) [123,124].

In contrast to absence of benefit, cervical cerclage demonstrated potential harm. A Cochrane review (5 RCTs, n = 612) found history-indicated cerclage increased preterm birth before 35 weeks: RR 2.15 (95% CI 1.15–4.01; NNH 6) [125,126]. Pessary in unselected twins (ProTWIN) showed no benefit (RR 0.98, 95% CI 0.69–1.39) [104].

Current guidelines recommend against progesterone, pessary, and cerclage in multiple gestations based on high-quality evidence demonstrating lack of benefit (progesterone, pessary) or potential harm (cerclage) [127]. Table 4 provides comprehensive intervention evidence in multiple gestations.

## 10. Implementation and Future Directions

### 10.1. Universal Cervical Length Screening

Universal cervical length screening programs represent one of the most significant opportunities for improving population health outcomes through early identification of cervical insufficiency and timely implementation of evidence-based interventions [129,130]. Cost-effectiveness analyses from multiple healthcare systems support the economic viability of universal screening programs [131,132].

Successful implementation requires comprehensive program development including standardized measurement protocols, training programs for sonographers and clinicians, and quality assurance measures [133,134]. Electronic health record integration, automated result reporting, and clinical decision support systems can facilitate implementation [135].

### 10.2. Biomarker Development

The development of biomarkers for improved risk stratification and treatment selection represents one of the most promising areas for advancing cervical insufficiency management [136,137]. Current research investigates inflammatory markers, cervical fluid biomarkers, genetic polymorphisms, and proteomics signatures that may enable more precise identification of patients at risk [138,139].

Integration of multiple biomarker types through machine learning and artificial intelligence approaches may provide more accurate risk prediction than individual markers alone [140,141]. These computational approaches can identify complex patterns and interactions among multiple variables [142].

### 10.3. Novel Therapeutic Approaches

Research into novel therapeutic approaches targeting different aspects of cervical insufficiency pathophysiology may expand treatment options beyond current interventions [143,144]. These investigations include matrix metalloproteinase inhibitors, anti-inflammatory agents, advanced drug delivery systems, and regenerative medicine approaches [145,146].

## 11. Clinical Algorithm and Decision-Making

**Figure 1:**
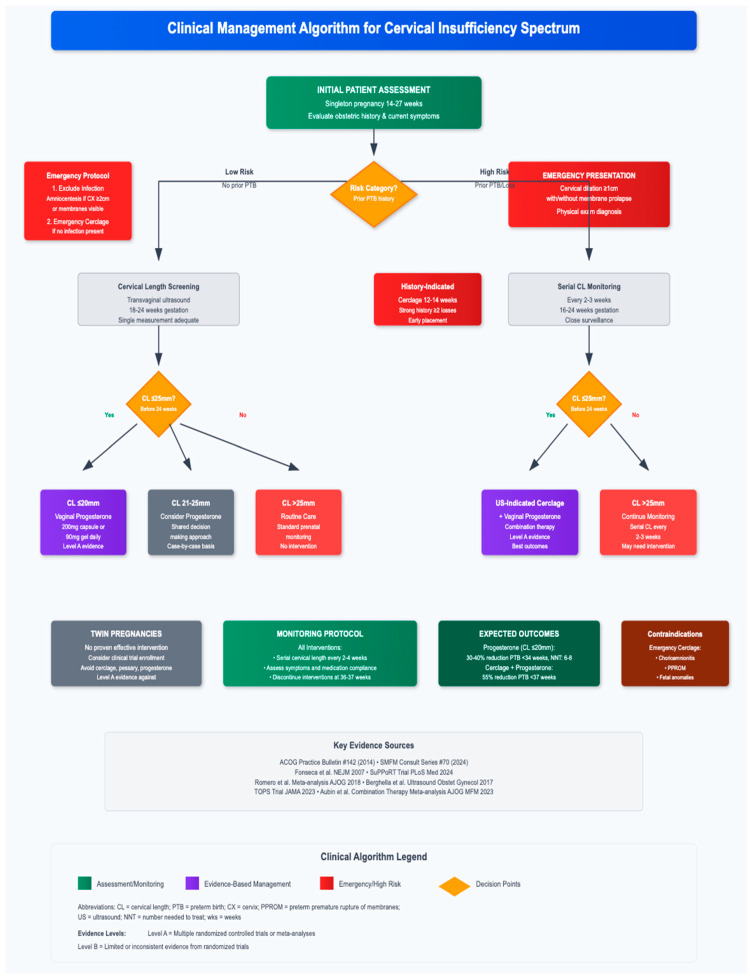
Clinical Management Algorithm for Cervical Insufficiency Spectrum.

Figure 1 presents a comprehensive clinical management algorithm for the spectrum of cervical insufficiency presentations, integrating the evidence-based diagnostic and therapeutic approaches discussed throughout this review. This algorithm provides a systematic framework for clinical decision-making from initial patient assessment through definitive management strategies, based on current professional society guidelines and landmark clinical trials [1,53,54].

The algorithm begins with initial patient assessment in singleton pregnancies at 14–27 weeks’ gestation, evaluating obstetric history and current symptoms to stratify patients into appropriate risk categories. This risk stratification forms the foundation for subsequent management decisions and has been validated in multiple cohort studies [3,4,14].

For low-risk patients without prior spontaneous preterm birth, the pathway emphasizes cervical length screening with transvaginal ultrasound at 18–24 weeks’ gestation, following international practice guidelines [53,60,61]. When cervical length is found to be ≤25 mm, management branches are based on specific measurements: cervical length ≤20 mm receives vaginal progesterone therapy (200 mg daily or 90 mg gel) with Level A evidence supporting this intervention [76,77,78]. Patients with cervical length 21–25 mm may receive progesterone based on shared decision-making approaches and individualized clinical assessment [53,99,100]. Those with cervical length >25 mm continue with routine prenatal care and standard monitoring protocols.

High-risk patients with prior spontaneous preterm birth before 37 weeks or classic history of cervical insufficiency follow distinct management pathways. Those meeting criteria for history-indicated cerclage, defined as two or more consecutive second-trimester losses (14–27 weeks) or extremely preterm births before 28 weeks with characteristic painless cervical dilation, receive prophylactic cerclage placement at 12–16 weeks’ gestation [1,91,92]. This approach demonstrates 70–90% success rates in appropriately selected patients [93].

Alternatively, serial cervical length monitoring every 2–3 weeks from 16–24 weeks’ gestation identifies high-risk patients requiring ultrasound-indicated cerclage when cervical shortening ≤25 mm is detected [85,86,87]. This precision medicine approach achieves outcomes comparable to prophylactic cerclage while avoiding unnecessary procedures in approximately 60% of patients who maintain normal cervical length [89,90]. Evidence supports consideration of combination therapy with cerclage plus vaginal progesterone in patients with very short cervical length, demonstrating superior outcomes compared to either intervention alone [96,97].

Emergency presentations with cervical dilation ≥1 cm and effacement ≥50% at 14–27 weeks without adequate uterine contractions, often with visible membrane prolapse, require immediate comprehensive assessment [90,107]. The critical pathway includes systematic infection exclusion through amniocentesis when cervical dilation exceeds 2–3 cm, examining amniotic fluid for microbiological and biochemical evidence of intraamniotic infection [112,113,114]. In the absence of contraindications including established infection, active vaginal bleeding, advanced labor progression, fetal death or lethal anomalies, emergency cerclage placement represents a last-resort intervention achieving 35–70% success rates depending on clinical presentation characteristics [108,115,116].

The algorithm incorporates special considerations for twin pregnancies, where current evidence demonstrates no proven effective intervention [120,121]. Strong recommendations against cerclage, pessary, and progesterone use in multiple gestations are based on high-quality evidence showing lack of benefit or potential harm [122,124,125,126,127].

Monitoring protocols are integrated throughout the algorithm, emphasizing serial cervical length assessments every 2–4 weeks for high-risk patients [67,68], systematic evaluation for signs and symptoms of preterm labor, and adherence monitoring for prescribed interventions. Patient education regarding warning signs of preterm labor and importance of medication compliance are essential components of comprehensive care [99,100].

Expected outcomes vary by intervention and patient characteristics. Vaginal progesterone (200 mg daily) in women with singleton pregnancy and cervical length ≤ 20 mm achieves 31% reduction in preterm birth before 33 weeks (RR 0.69, 95% CI 0.55–0.88; NNT = 14) [78]. Ultrasound-indicated cerclage demonstrates 30% reduction in preterm birth before 35 weeks (RR 0.70, 95% CI 0.55–0.89) [87]. Combined cerclage plus vaginal progesterone in women with prior preterm birth and cervical length < 25 mm shows 36% reduction in preterm birth before 35 weeks (RR 0.64, 95% CI 0.42–0.97; NNT 6), most pronounced when cervical length < 15 mm (post hoc analysis) [96,97].

The algorithm emphasizes individualized decision-making based on comprehensive risk assessment, patient preferences, and clinical circumstances, consistent with contemporary shared decision-making frameworks [99,100]. Recent comparative effectiveness research demonstrating equivalent outcomes among different interventions in appropriately selected populations supports this flexible, patient-centered approach [98].

Implementation of this clinical algorithm requires systematic attention to quality assurance measures, standardized assessment protocols, and multidisciplinary coordination to optimize outcomes [129,130,131,132]. Electronic health record integration and clinical decision support systems can facilitate consistent application of evidence-based management strategies across different healthcare settings [133,134,135].

This comprehensive algorithm synthesizes the complex decision-making required across the spectrum of cervical insufficiency, providing clinicians with a practical tool for implementing evidence-based strategies while maintaining necessary flexibility for individual patient circumstances. The systematic approach from initial assessment through definitive management reflects current best practices supported by robust clinical evidence and professional society recommendations [1,53,54,55,56].

## 12. Conclusions

Cervical insufficiency management has undergone transformation over recent decades, evolving from empirical approaches to evidence-based strategies grounded in rigorous scientific research. Contemporary understanding recognizes cervical insufficiency as a complex, multifactorial syndrome existing along a clinical spectrum, enabling more nuanced and individualized approaches to patient care.

The establishment of evidence-based diagnostic frameworks incorporating history-based, ultrasound-based, and physical examination categories provides clinicians with clear guidance while maintaining necessary flexibility for patient-specific considerations. The therapeutic landscape has been revolutionized by robust evidence demonstrating the effectiveness of vaginal progesterone therapy and cervical cerclage interventions in appropriately selected patients.

Recent landmark comparative effectiveness research has challenged traditional intervention hierarchies and demonstrated equivalent outcomes among different therapeutic approaches in appropriately selected patients. These findings emphasize that patient selection factors, clinical expertise, and individual circumstances may be more important determinants of success than specific intervention choice, supporting flexible and personalized treatment strategies.

Multiple gestations represent one of the most significant remaining challenges, with consistent failure of singleton pregnancy-derived interventions to demonstrate benefit. This pattern underscores the distinct pathophysiology of preterm birth in multiple pregnancies and emphasizes the critical importance of pregnancy-type-specific research and evidence-based guidelines.

Future research directions encompass biomarker development for personalized risk assessment, novel therapeutic approaches targeting specific molecular pathways, and advanced monitoring technologies that may enable more precise intervention timing. The collaborative efforts of researchers, clinicians, patients, and healthcare systems remain essential for addressing remaining challenges and optimizing outcomes for patients affected by this complex condition.

## Figures and Tables

**Table 1:** Comprehensive Diagnostic Framework for Cervical Insufficiency.

Diagnostic Category	Specific Clinical Criteria	Cervical Length Threshold	Evidence-Based Management	Evidence Level	Typical Success Rate
History-Based [1,5,53,54]	≥2 consecutive second-trimester losses (14–27 weeks) or extremely preterm births <28 weeks with painless cervical dilation and minimal contractions	Not applicable	History-indicated cerclage at 12–16 weeks	Grade B	70–90%
Ultrasound-Based [10,53,54,55,57,58]	Previous spontaneous preterm birth <37 weeks + current pregnancy cervical length ≤25 mm before 24 weeks	≤25 mm	Ultrasound-indicated cerclage ± progesterone	Grade A	75–85%
Physical Examination-Based [1,53,54]	Cervical dilation ≥1 cm and effacement ≥50% at 14–27 weeks without adequate uterine contractions	Variable with advanced dilation	Emergency cerclage after infection exclusion	Grade C	35–70%
Asymptomatic Short Cervix [10,53,54,55]	Incidental finding on routine screening, no history of spontaneous preterm birth <37 weeks	≤20 mm (definitive) 21–25 mm (discretionary)	Vaginal progesterone with serial monitoring	Grade A	65–80%

**Table 2:** Comparative Effectiveness in High-Risk Patients.

Intervention Strategy	Patient Population	Preterm Birth <34 Weeks	Preterm Birth <32 Weeks	Relative Risk (95% CI)	Number Needed to Treat	Major Complications
Ultrasound-Indicated Cerclage [87]	Prior PTB < 37 weeks + CL ≤ 25 mm before 24 weeks	15–18% vs. 26–30% *	8–12% vs. 18–22% †	0.70 (0.55–0.89)	8	2–5%
History-Indicated Cerclage [93]	≥2 s-trimester losses or PTB < 28 weeks	10–15% vs. 25–35% ‡	5–8% vs. 15–20%	0.55 (0.40–0.75)	5	3–8%
Combined Cerclage + Progesterone [96,97]	High-risk (prior PTB or CL ≤ 25 mm)	11% vs. 24% §	6% vs. 15%	0.45 (0.29–0.69)	8	2–6%
Progesterone Alone [78]	CL ≤25 mm, no prior PTB	18–22% vs. 28–35%	10–14% vs. 20–25%	0.70 (0.55–0.90)	9	<1%

PTB, preterm birth; CL, cervical length; CI, confidence interval. Preterm birth rates shown as intervention group vs. control/comparison group. Number needed to treat calculated to prevent one preterm birth before 34 weeks of gestation. Major complications include preterm premature rupture of membranes, chorioamnionitis, and procedure-related complications. * Based on Berghella et al. meta-analysis [87]: 5 RCTs with individual patient data from 504 women. Primary outcome PTB < 35 weeks: 28.4% (71/250) cerclage vs. 41.3% (105/254) no cerclage; RR 0.70 (95% CI 0.55–0.89). † Based on MRC/RCOG multicentre RCT [91]: PTB < 33 weeks 13% cerclage vs. 17% control in high-risk subgroup. ‡ From Aubin et al. meta-analysis [96]: Combined therapy showed PTB < 37 weeks RR 0.51 (95% CI 0.37–0.79) vs. cerclage alone and RR 0.75 (95% CI 0.58–0.96) vs. progesterone alone. Supported by Tolosa et al. [97] multicenter cohort. § Based on Fonseca et al. NEJM trial (2007;357:462-9): PTB < 34 weeks 19.2% progesterone vs. 34.4% placebo (RR 0.56; 95% CI 0.36–0.86). Extended to ≤25 mm threshold based on Romero et al. individual patient data meta-analysis [78] showing consistent benefit across cervical length range with 44% reduction in PTB < 33 weeks (RR 0.62; 95% CI 0.47–0.81). *Evidence Quality: Ultrasound-indicated cerclage and vaginal progesterone supported by Grade A evidence from multiple RCTs and individual patient data meta-analyses. History-indicated cerclage supported by Grade B evidence. Combined therapy represents emerging evidence (Grade B-C) from predominantly observational studies.*

**Table 3:** Emergency Cerclage Outcomes Stratified by Clinical Characteristics.

Clinical Presentation Factor	Successful Pregnancy Prolongation (%)	Mean Gestational Age at Delivery (Weeks)	Neonatal Survival Rate (%)	Major Maternal Complications (%)
Cervical Dilation 2–3 cm [115]	75–85	32–34	80–90	5–10
Cervical Dilation 4–6 cm [115]	45–60	28–30	60–75	10–15
Visible Membrane Prolapse [116]	35–50	26–28	50–65	15–25
Negative Amniocentesis [113]	70–80	31–33	80–85	5–12
Positive Amniocentesis [113]	20–30	24–26	30–40	20–35

**Table 4:** Intervention Evidence in Multiple Gestations.

Intervention Type	Key Studies	Total Sample Size	Primary Outcome	Result Summary	Relative Risk (95% CI)	Current Recommendation	Evidence Quality
Vaginal Progesterone	PREDICT trial [122]	500+ twin pregnancies	PTB < 34 weeks	No significant benefit	1.04 (0.79–1.37)	Not recommended	High
17-OHPC Injections	Rouse et al. [124]	661 twin pregnancies	PTB < 35 weeks	No significant benefit	0.99 (0.81–1.21)	Not recommended	High
Cervical Pessary	ProTWIN trial [128]	813 twin pregnancies	Composite poor outcome	No significant benefit	1.02 (0.84–1.24)	Not recommended	Moderate
Cervical Cerclage	Meta-analyses [125,126]	200+ twin pregnancies	PTB < 34 weeks	Potential harm	1.23 (0.98–1.54)	Contraindicated	Moderate

PTB = preterm birth; 17-OHPC = 17-alpha hydroxyprogesterone caproate.

## Data Availability

No new data were created or analyzed in this study. Data sharing is not applicable to this article.
